# Alkalies in the Treatment of Hooping-Cough

**Published:** 1847-02

**Authors:** 


*Alkalies in the Treatment of Hooping-Cough.*—Dr. Allnat, of London, attributes the spasmodic action of the glottis, which occurs after the febrile action has somewhat subsided, to the presence of acid in the stomach; to relieve which alkalies—ammonia, carb. potass, &c.—are advised. He says:

After preliminary purgation with calomel, (conjoined with antimony, if the febrile symptoms run high,) and an occasional emetic to clear the stomach, nothing in my experience is so efficacious as small and repeated doses of the carbonate of potassa. The following combination has been extensively distributed to the poor in seasons when hooping-cough has raged as an epidemic, and I can attest the almost invariable success which has attended its administration—what portion of the merit is due to the cochineal I do not know:—Take of carbonate of potassa, one drachm; cochineal, ten grains; boiling water, half a pint. For an infant, one teaspoonful to be taken thrice daily, the dose increased according to age. *London Lancet.*

